# Supplementary material for: The Human Gut Microbiome Activity Is Resilient and Stable for up to Six Months: A Large Stool Metatranscriptomic Study
Source: Microorganisms. 2026 Apr 7;14(4):835. doi: 10.3390/microorganisms14040835 (PMC13118932; doi:10.3390/microorganisms14040835)
Supplement: Supplementary file 1 [file microorganisms-14-00835-s001.zip › microorganisms-4202532-supplementary.pdf]

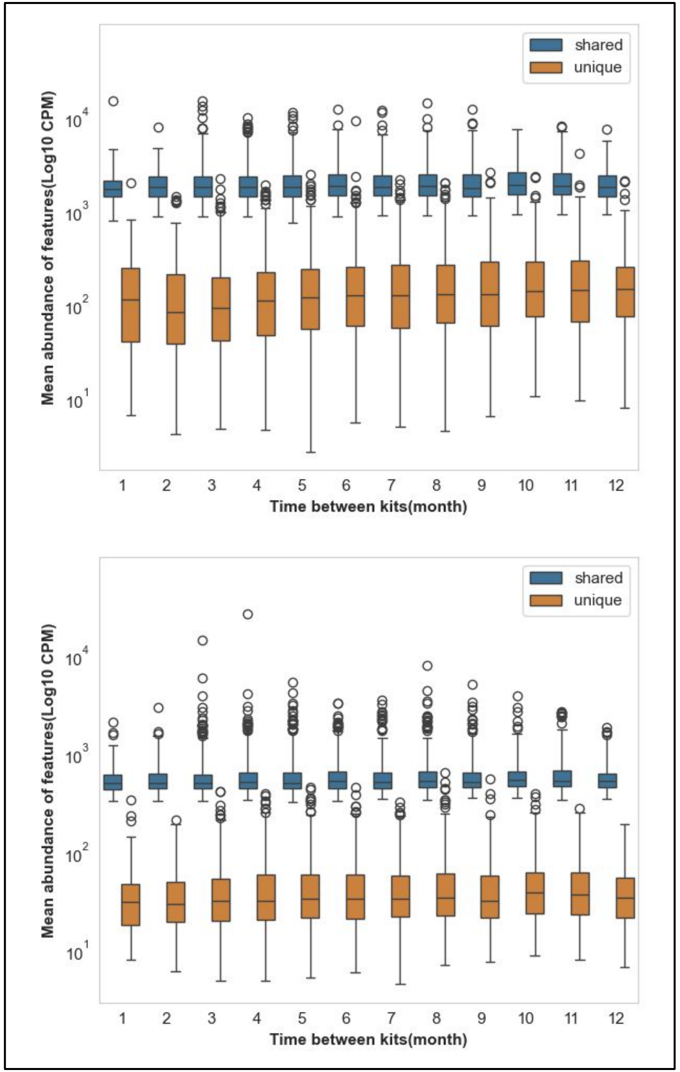

Supplemental Figure S1.
Mean abundance of shared and unique features, comparing each baseline to monthly sample. Shared (blue) and unique (orange) features for species (a) and KOs (b). All months show a significantly ($p<0.05$) increased abundance of the shared features compared to the unique features.

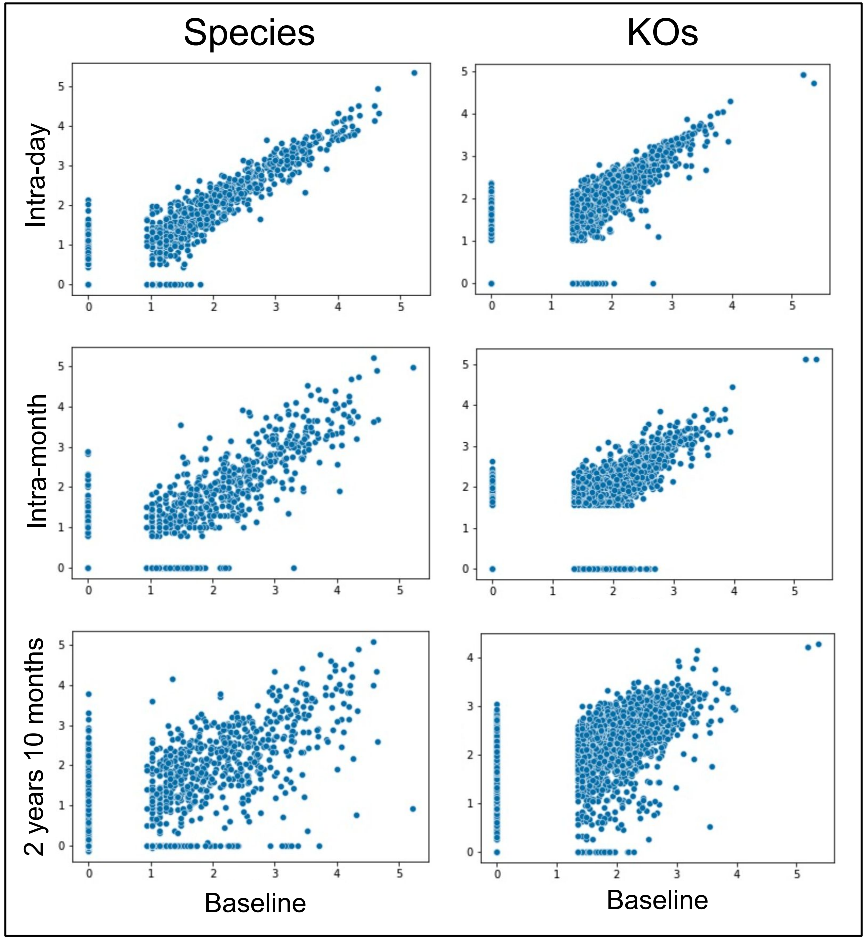

Supplemental Figure S2.

Composition and functional profiles for donor 1. Donor 1 species (left) and KOs (right). The compositional (species) and functional (KO) profiles of the gut microbiome for donor 1 are plotted, normalized to log10(CPM) (Counts Per Million), showing a drift over time. Despite the drift, the gut microbiome retains a significant correlation even after almost three years.

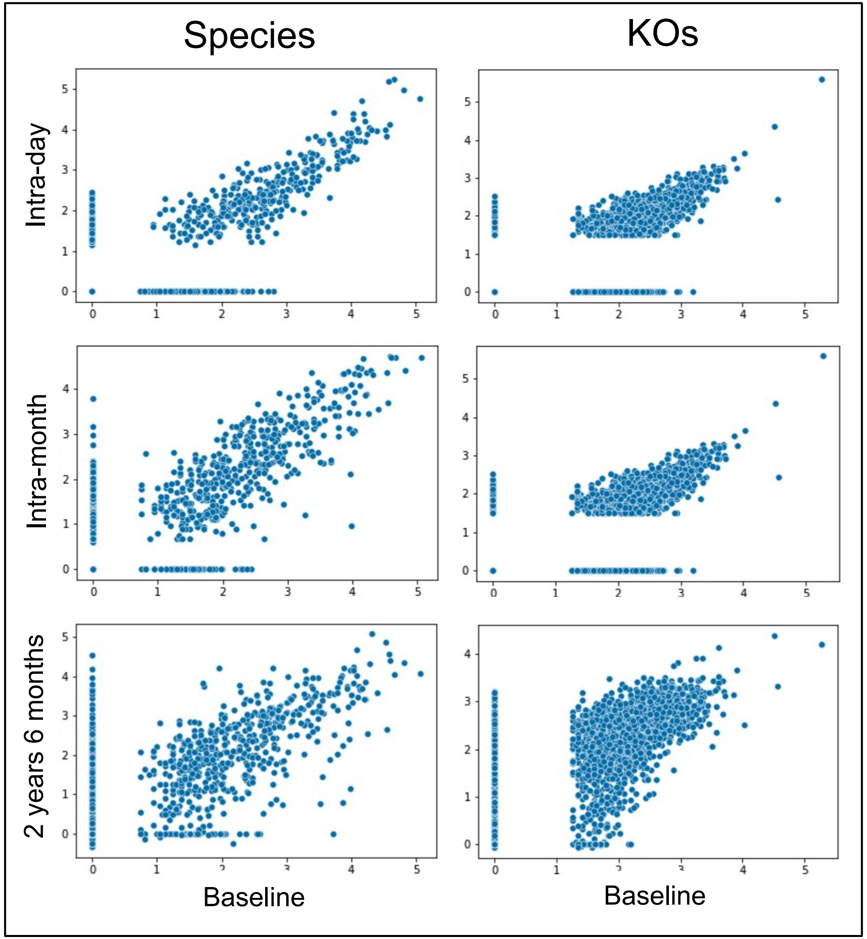

Supplemental Figure S3.
Composition and functional profiles for donor 2. Donor 2 species (left) and KOs (right). The compositional (species) and functional (KO) profiles of the gut microbiome for donor 2 are plotted, normalized to log10(CPM) (Counts Per Million), showing a drift over time. Despite the drift, the gut microbiome retains a significant correlation even after almost three years.
